# Key Formulation Variables in Tableting of Coated Pellets

**DOI:** 10.4103/0250-474X.45391

**Published:** 2008

**Authors:** V. S. N. Murthy Dwibhashyam, J. Vijaya Ratna

**Affiliations:** Department of Pharmaceutical Sciences, Andhra University, Visakhapatnam-530 003, India; 1 TherDose Pharma Pvt. Ltd, Plot No: 30-32, Survey No: 400, 1st Floor, Prashanth Nagar, IE, Kukatpally, Hyderabad-500 072, India

**Keywords:** Multiple unit dosage forms, pellets, coating, cushioning, compression

## Abstract

Multiple unit controlled release dosage forms offer various advantages over their single unit counterparts. Most of these advantages are associated with the uniform distribution of multiparticulates throughout the gastrointestinal tract. Though coated pellets can be filled into hard gelatin capsules, tablet formulation is the preferred one because of various advantages associated with it. However, compression of coated pellets is a challenging task necessitating the optimization of various formulation and process variables. The key formulation variables include composition, porosity, size, shape and density of the pellets; type and amount of polymer coating; nature, size and amount of tableting excipients. The pellet core should be strong with some degree of plasticity. It should be highly porous, small, with an irregular shape. The critical density to achieve prolonged release was reported to lie between 2.4 and 2.8 g/cm^3^. Acrylic polymer films are more flexible and more suitable for the coating of pellets to be compressed into tablets. Thicker coatings offer better resistance to frictional forces. Solvent based coatings are more flexible and have a higher degree of mechanical stability than the aqueous based ones. The tableting excipients should have cushioning property. They should not be significantly different in size and density from those of the pellet cores in order to avoid segregation. Addition of 30%-60% of tableting excipients is necessary to avoid any damage to the polymer coat and to retain its functional property.

Extended release and delayed release dosage forms comprising multiple units such as pellets offer various advantages over single unit dosage forms such as coated tablets and capsules^1^. The major advantages are reduced risk of local irritation and toxicity, less variable bioavailability, reduced inter and intra-individual variations in bioavailability caused for example by food effects, reduced premature drug release from enteric coated dosage forms in the stomach because of a more rapid transit time of coated pellets when compared to enteric coated tablets, and various drug release profiles can be obtained by simply mixing pellets with different release characteristics. Most of these advantages are associated with the uniform distribution of multiparticulates throughout the gastrointestinal tract. The drawbacks of multiple unit modified release dosage forms are that their manufacture is technically more complicated, time-consuming and expensive.

With respect to the final dosage form, the coated pellets can either be filled into hard gelatin capsules or can be compressed into tablets. However, there are some disadvantages with capsules such as feasibility of tampering, difficulties in esophageal transport, and higher production costs. Therefore, tablet formulation is the preferred final dosage form. Only a few multiple unit containing tablet products are available such as Beloc® ZOK, and Antra MUPS®^2^. This is due to the inherent challenges involved in the compression of coated pellets. Ideally, the compacted pellets should disintegrate rapidly into individual pellets in gastrointestinal fluids and the drug release pattern of the coated pellets should not be affected by the compression compress. Certain formulation and process parameters play an important role in successful production and functioning of the multiple unit-containing tablets. This article reviews those key formulation variables.

## PELLET CORE

Properties of the pellets such as composition, porosity, size, and density have been reported to affect the functioning of the multiple-unit containing tablets. An understanding of the compression behaviour of uncoated pellets can provide a basis for the formulation of multiple unit tablets.

### Composition of the pellets:

Pellets have been shown to behave differently on compaction and consolidation than powder of the same material^3^. Wang *et al*. found that the compactibility of lactose-rich pellets was better than that of MCC-rich pellets and the poor compactibility of the later was ascribed to the loss of plasticity of MCC during the wet granulation process^4^. The desirable mechanical properties of the core are that they should be strong, should not be brittle and have a low elastic resilience^5^.

Beckert and co-workers studied the behaviour of the enteric-coated bisacodyl pellets on compression. They compacted coated pellets of two different crushing strengths with different excipients, and concluded that the harder pellets were better able to withstand compression forces as they deformed to a lesser degree^6^. The core should have some degree of plasticity, which can accommodate changes in shape and deformation during compression^7^. It should deform and recover after compression without damage to the coating. Therefore, the core should preferably contain a material that undergoes plastic deformation during compression. Microcrystalline cellulose is such an excipient.

The compression behaviour of granulated microcrystalline cellulose has been thoroughly studied by Johansson^8^. Maganti and Celik compared the compaction behaviour of pellet formulations, mainly consisting of MCC, to that of the powders from which they were formed and found significant differences between the two^9^. The powders were found to compact by plastic deformation and produced strong compacts, while the pellets exhibited elastic deformation and brittle fragmentation, resulting in compacts of lower strength. This was ascribed to the low surface to volume ratio of the granules, which might result in a decreased area of contact between the particles as they consolidate.

The compression behaviour of pellets consisting of MCC alone or in combination with excipients such as polyethylene glycol and dicalcium phosphate was studied by Nicklasson^10^. The possibility of using pellets formed mainly of a hard, low deformable material (dicalcium phosphate) with different porosities was studied by Nicklasson *et al*. They concluded that the pellet fragmentation is minimal for pellets of both high and intermediate porosity *i.e*., the pellets deform and probably become denser but remain as coherent units without significant flaws in their structure during the compaction process^11^.

Incorporating a soft waxy material in the powder mixture from which the pellets are formed can modulate the compression behaviour of pellets. Nicklasson and Alderborn studied the tabletting behaviour of MCC pellets containing PEG 6000. They found an increase in the deformation propensity of the pellets by the incorporation of PEG. However, the character of the deformation behaviour changed towards an increased tendency for local deformation during compression *i.e.*, the ability of adjacent pellet surfaces to conform to each other increased, without marked changes in the main dimensions and porosity of the pellets^12^.

Santos *et al.*^13^ studied the compaction behaviour of xanthan gum pellets of different compositions containing one of three different fillers *viz*., lactose monohydrate, tribasic calcium phosphate and β-cyclodextrin at 16% w/w and xanthan gum, also at 16% w/w. They concluded that permanent deformation was the most relevant mechanism involved in the compression of these units. Fragmentation was very minimal and densification was considered as a significant mechanism of compression^13^. Iloanusi and Schwartz found MCC based bead formulations incorporating wax to be more compressible than those made without wax. As the amount of wax increased, plastic deformation became the predominant deformation mechanism, while compacts without wax underwent higher elastic recovery^14^.

Eudragit RS PO and RL PO have been shown to be beneficial as core materials in the production of plastic pellets. In a recent study by Abbaspour *et al.*^15^ various ibuprofen pellet formulations were prepared by extrusion-spheronization that differed in the % ibuprofen (40, 60, and 80) and % Eudragit RS PO/RL PO (0, 50, and 100) with 3% w/w PVP K30 and 10% w/w Avicel PH 101. All pellet formulations were cured in oven at 60° for 24 h. It was shown that the cured pellets with 40% or 60% drug underwent plastic deformation without any fracture under mechanical tests viz., crushing strength or yield point and elastic modulus. Plastic behaviour of these cured pellets was also evident from the scanning electron microscopy (SEM) of the compressed pellets. Pellets with 80% drug exhibited brittle properties even after curing because of the absence of proper amount of Eudragit in their structure. It was shown that the structural deformation of the Eudragit polymers from glassy state to rubbery state upon curing was responsible for the observed plastic behaviour of the cured pellets. The polymeric structural changes were evident by DSC studies. These results revealed that thermal treatment of Eudragit based pellets could be advantageous in the production of plastic pellets that are intended to be coated and compressed into tablets^15^.

### Porosity of the pellets:

Porosity of the pellets is another key factor that affects the compaction pattern and thereby affects the polymer coat integrity during compression. Tuton and co-workers^16^ studied the compaction behaviour of pellets of three different porosities, containing microcrystalline cellulose and salicylic acid that were prepared by extrusion-spheronisation and coated with ethyl cellulose. They found that the coating did not significantly interfere with the compression behaviour of the pellets. The effect of intragranular porosity on the compression behaviour of and drug release from the reservoir pellets was high; compacted pellets of high porosity were highly densified and deformed, while drug release was unaffected, whereas for compacted low porosity pellets the drug release rate was markedly increased while there was only slight densification and deformation. It can be inferred from this study that the use of highly porous pellets was advantageous, in terms of preserving the drug release profile after compaction, compared with pellets of low porosity^16^.

The compression behaviour and compactibility of nearly spherical microcrystalline cellulose pellets of different porosities and mechanical properties was investigated by Johansson *et al*^17^. The pellet porosity was found to control the degree of deformation that the pellets underwent during compression. Thus, the degree of deformation of the pellets, caused by a reposition of primary particles within the pellet, seemed to be controlled by the total volume of air that surrounds the primary particles in the pellets. An increase in pellet porosity increased the degree of deformation of the pellets during compression and the tensile strength of the tablets because of the formation of stronger intergranular bonds.

Nicklasson *et al.* studied the compaction behaviour of pellets prepared from 4:1 mixtute of dicalcium phosphate and microcrystalline cellulose with porosities in the range of 26-55%. The pellet porosity was found to significantly affect the tableting behaviour of the DCP/MCC pellets. Increasing the pellet porosity increased the compressibility of the pellets throughout the whole range of compaction pressure used which can be attributed to an increased degree of deformation and densification of individual pellets with increased porosity. The presence of DCP made the pellets less compressible during compaction and the pore structure of the tablets more closed. It is inferred that when the primary particles are harder, it will be more difficult for them to flow within the pellet and the pellets will thus be more rigid and less prone to deform and densify during compression. However, the more rigid nature of the pellets leads to a change in mode of deformation during compaction, from bulk deformation towards surface deformation^18^.

The relationship between pellet porosity and the compaction behaviour was further confirmed through a study of drying rate effect on porosity and tabletting behaviour of microcrystalline pellets^19^. An increased drying rate gave more porous pellets, due to decreased pellet densification during the drying process which were more deformable and which formed tablets of a higher tensile strength.

### Size of the pellets:

The size of the pellets also affects the compaction properties and the drug release from the compacted pellets. At the same coating level, smaller pellets were more fragile than larger pellets. This was attributed to the reduced film thickness of the smaller pellets because of the larger surface area^20^. Small pellets have been found to be less affected than larger pellets by the compaction process. Haslam *et al*. correlated this to the individual bead strength *i.e.,* the smaller beads were significantly stronger, relative to their size, than the larger ones^21^.

Ragnarson *et al*. found that increasing the particle size resulted in more damage to the coating, as indicated by larger differences between the release profiles of compressed and uncompressed pellets^22^. Johnson *et al*. found the deformation of individual pellets to be correlated to their size *i.e.,* larger pellets were more readily deformed. This was explained by a reduced number of force transmission points with increasing size of the pellets and therefore an increased contact force on each pellet, resulting in a higher degree of deformation. Similar kind of observations were reported in another study carried out by Nicklasson *et al*^23^.

### Shape of the pellets:

Shape of the pellets was found to have a bearing on the compression behaviour and tablet forming ability of granular materials formed from microcrystalline cellulose. A change in granule shape towards a more irregular shape induced a more complex compression behavior of the granules *i.e.*, primarily attrition of the granules was induced. A more irregular shape increased the bed voidage, which allowed an increased degree of deformation that the granules underwent during compression.

The shape-induced increased degree of granule deformation during compression resulted in tablets of a more closed pore structure and a higher tensile strength. An irregular shape and a rougher surface texture made the granules less sensitive to lubrication in terms of their compactibility. This was possibly the result of a rupture of the lubricant film due to deformation or attrition during compression, or of an incomplete surface coverage of the granules by the lubricant before compression^24^.

### Density of the pellets:

Density of pellet is of particular importance especially if it is required to achieve prolonged gastric residence. Clarke *et al*. investigated the comparative gastrointestinal transit of pellet systems of density 1.5, 2.0 and 2.4 g/cm^3^ and found no difference in gastrointestinal transit time^25^. Devereux *et al.* compared the gastrointestinal transit time of a multiple unit formulation of density 2.8 g/cm^3^ with the same of a control formulation of density 1.5 g/cm^3^ and found significantly delayed gastric emptying of the heavier formulation in both fed and fasted conditions^26^. Therefore, it can be concluded that the critical density to achieve prolonged gastric residence may lie between 2.4 and 2.8 g/cm^3^.

Achieving content and weight uniformity is another challenging formulation task associated with tabletting of multiple units. Density and size of the pellets play an important role in this regard. If pellets are compressed with excipients of smaller particle size and lesser density, weight variation occurs because of segregation. This problem can be solved if pellets with a narrow size distribution are compressed together with excipients of similar size, shape and density^27^.

Disintegrating tablets containing particles of about 1mm diameter need to be compressed with a certain amount of excipient, otherwise the mixture will not form stable tablets, and cracking will occur in the film coatings on the pellets^6^. Beckert *et al*. compressed pellets of about 1 mm diameter to tablets of 10 mm diameter. Compression trials were carried out with 10%, 30%, 50% and 70% w/w pellets. A good uniformity of mass and content was obtained only with 50% w/w pellets occupying 30% v/v. It was ascribed to the formation of a percolating cluster of coarse material. If less than 30% v/v pellets were compressed, suitable granules had to be added until 30% v/v was reached to form a percolating cluster. Filler-binders for direct compression, like Avicel PH 200, can be used in tablet manufacture if a percolating cluster of the coarse components is ensured. The particle size and density of these excipients should not differ too much from that of the coarse components, *i.e.*, the pellets or granules^28^.

## POLYMER COATING

The nature of the polymer, type and amount of polymer coating have significant impact on the compression-induced changes in the film coating structure.

### Nature of the polymer and polymer coating:

Polymers used in the film coating of solid dosage forms fall into two broad groups; cellulosic polymers and acrylic polymers^29^. The utility of hydroxypropyl methylcellulose (HPMC E15) as a controlled release film was investigated by Sadeghi *et al*^30^. It was found that the drug release from pellets coated with HPMC E15 (up to 20% w/w) was fast and completed within 1 h.

Many of the polymers used for controlled release have been formulated into aqueous dispersions so as to overcome the disadvantages associated with the use of organic polymer solutions. The polymer coating should be highly elastic and flexible to be able to adapt to the deformation of the pellets without rupturing^5^,^31^. Aulton and co-workers found that the films exhibiting a relatively high elastic modulus and apparent Newtonian viscosity provide the highest protection to the pellet core and coating on compaction^5^. The polymer coat should not get ruptured during compression. It should have sufficient mechanical stability and should remain intact during compression in order to control the drug release.

Solvent based coatings have been found to be more flexible and have a higher degree of mechanical stability than aqueous-based ones, and therefore less affected by compaction. Both ethyl cellulose and methacrylate copolymers were investigated in this study. Ethyl cellulose films cast from the plasticized pseudolatexes, Aquacoat®, and Surelease® were very brittle and weak with low values of puncture strength and elongation (< 5%)^32^.

The compression of diltiazem HCl pellets coated with ethyl cellulose resulted in a faster drug release when compared to the release from noncompressed pellets^33^. The tensile properties of free films as a function of plasticizer were measured. The elongation varied between 0.93% and 4.28% for different plasticizers. This is too low to result in flexible films, which deform and do not rupture during compression. In another investigation by Bechard and Leroux, the use of ethyl cellulose aqueous dispersions (Aquacoat® and Surelease®) led to failure of the coating through the formation of cracks and flaws^20^.

When compared to ethyl cellulose films, the films prepared from acrylic polymers are more flexible and therefore more suitable for the coating of pellets to be compressed into tablets. The flexibility of various acrylic polymeric films was compared with respect to the elongation values^32^. Films of Eudragit® NE30D dispersion were very flexible with the elongation value in excess of 365%. With plasticized Eudragit® RS and RL30D, which are dispersions based on the cationic polymer, flexible films were obtained with elongation values in excess of 125%. The flexibility of enteric acrylic latex Eudragit® L30D was poor with elongation value of less than 1%.

In a study carried out by Dashevsky *et al*. the compression of pellets containing propranolol HCl and coated with aqueous ethyl cellulose dispersion, Aquacoat® ECD (plasticized with 25% w/w triethyl citrate) resulted in coating rupture. Plasticizer-free Kollicoat® SR coatings were too brittle and ruptured during compression. However, the addition of only 10% w/w triethyl citrate as a plasticizer improved the flexibility of the films and allowed compaction of the pellets^2^.

Normally, the ductility of aqueous-based coatings can be improved by the addition of plasticizers, but this is often accompanied by a reduction in the tensile strength^34^. In contrast, Felton and co-workers found the tensile strength of film coated beads to increase with increasing plasticizer content, and as the degree of plasticization of the polymer increased, the film coating became more elastic and was able to deform during compression^35^. Okarter and Singla investigated the effects of 6%, 12% and 18% of four plasticizers polyethylene glycol 400, propylene glycol, tributyl citrate and triethyl citrate on the release of metoprolol tartrate from granules coated with a Eudragit® RS30 film. The type and concentration of plasticizer were found to affect the drug release from the granules. The dissolution became slower with increasing concentration of plasticizer and the resulting improvement of the film^36^.

However, ways are being tried out to find aqueous polymer dispersions that give flexible coatings. Eudragit® NE30D and Kollicoat® EMM 30D have a low minimum film forming temperature (5°) and no curing effects have been reported. However, a higher tackiness during the coating process and during storage of the coated dosage form requires the addition of anti-tacking agents. Kollicoat® SR3OD is a new aqueous colloidal polyvinyl acetate dispersion used for extended release coatings. It is stable against sedimentation, has a low viscosity of about 54 mPa and a negative zeta potential of −23.2 mV because of the presence of the anionic surfactant, sodium dodecyl sulfate. It has low minimum film formation temperature of about 18°. Coated pellets showed no aging or curing effect.

The drug release from Kollicoat® SR3OD coated pellets was almost independent of the pH and ionic strength of release medium^37^. Sawicki and Lunio investigated the compressibility of floating pellets of verapamil hydrochloride coated with Kollicoat® SR30D dispersion. They found no difference between the release profile of the compressed and uncompressed pellets coated with Kollicoat dispersion at a film thickness of 50 μ having propylene glycol as plasticizer and stabilized with povidone^38^.

Though the coating of aqueous colloidal dispersions is gaining importance, it has certain inherent shortcomings. It can be sensitive to different factors such as temperature, pH, addition of electrolytes and other polymers, potentially resulting in the coagulation of the dispersions. The film formation process is fundamentally different for the two coating techniques. In organic solvent-based systems, the polymer solutions undergo sol-to-gel transitions upon solvent evaporation to finally form the polymeric films. Upon spraying aqueous polymer dispersions, the polymer particles are deposited on the surfaces of the solid dosage forms. The colloidal particles come into direct contact with each other and form close packed arrays due to water evaporation and the interfacial tension between water and polymer.

Lecomte *et al*. studied the effects of the type of coating technique (aqueous v/s-organic) on drug release. Propranolol HCl-loaded pellets were coated with blends of ethyl cellulose and Eudragit® L. The drug release was strongly dependent on the type of coating technique. Both the slope and shape of the release curves were affected, indicating changes in the underlying drug release mechanisms. The observed effects were ascribed to the higher mobility of the macromolecules in organic solutions compared to aqueous dispersions, resulting in higher degrees of polymer-polymer interpenetration and thus, tougher and less permeable coatings^39^.

### Amount of polymer coating:

The amount of coating has its own role in protecting the polymer film integrity during compression. In general, a thicker coating can withstand damage better than a thinner one^27^. Beckert and co-workers found that the elasticity improves with the coating thickness of elastic coatings^6^. In an investigation by Wagner *et al*. however, it was found that the coating must be of at least a specific lowest thickness for the elasticity to have a synergistic effect on reduction of the coating damage during compaction^40^. In this study it was concluded that thicker coatings offer better resistance to frictional forces, and consequently cracks that are introduced into the coating during compression. Similar conclusion was drawn from a study conducted by Sawicki and Lunio in which, Verapamil hydrochloride cores containing sodium hydrocarbonate (20%), Avicel® PH 101 (10%), Arbocel® P 290 (33.4%), lactose (12.3%) and povidone K-30 (4.3%) were prepared by wet granulation and spheronization process. The pellets were coated with Kollicoat SR 30D using propylene glycol as a plasticizer at different thickness values viz. 35 and 50 μm. Compression studies of these pellets revealed that pellets with film thickness of 35 μm deformed on compression with considerably faster drug release. However, increasing film thickness to 50 μm could prevent the deformation caused by compression to a significant extent^38^.

## TABLETING EXCIPIENTS

It has been found that coated pellets can be compressed into tablets whilst retaining controlled release of the drug, provided that the effect of excipients and the compression force is considered and determined^41^. Several excipients have to be used to assist the compaction process and to prevent the rupture and damage of the coated pellets during compression. When reservoir pellets are compacted without including any excipients, disintegration of the tablets cannot be ensured and matrix tablets often form^42^,^43^.

### Nature of the excipients:

The ideal filler materials should prevent the direct contact of the pellets and act as cushion during compression. The theoretic void space of a powder of uniform spheres in closest packing is 26%. The filler materials must fill this void space as to prevent adhesion and fusion of the coated pellets during compression. The filler excipients can be either primary powder particles or can be in the form of secondary agglomerates, such as granules or pellets. The use of agglomerates is preferred, however, to reduce the risk of segregation owing to difference in particle size^44^.

The protective effect of an excipient depends on the particle size and the compaction characteristics of the material. In general, materials that deform plastically, such as microcrystalline cellulose and polyethylene glycol, give the best protective effct^45^. Though microcrystalline cellulose can be used as it is, due to particle size differences, segregation may be encountered resulting in weight variation and content uniformity problems. Granules produced by dry or wet granulation techniques having a similar size of the drug-loaded beads are able to minimize the segregation due to size similarities. However, the dry or wet granulation of microcrystalline cellulose containing mixtures decreases their compactibility^46^. The addition of brittle materials such as dicalcium phosphate and lactose was found to make the microcrystalline cellulose beads very hard, which are not easily deformed or broken.

The production of softer inert cushioning beads containing microcrystalline cellulose was not successful when water was used as the granulating agent. Replacement of part of the granulating solution with a polar organic solvent produced microcrystalline cellulose granules which are capable of cushioning controlled release particles and barrier coated particles from the compression forces used in tableting, thereby maintaining the physical integrity of the components of the tablet^47^.

Habib *et al*. prepared beads containing different MCC/lactose ratios and different types and levels of superdisintegrants by extrusion-spheronization followed by freeze-drying and found that the formulations containing high levels of MCC exhibited lower yield values, and thus were more compressible, than those containing high levels of lactose. The freeze-dried beads were found to exhibit both plastic deformation and some brittle fracture. However, the utility of these beads is yet to be evaluated practically by mixing and compacting them with drug-loaded membrane-coated sustained release beads^48^.

In an investigation by Remon and Paul, biologically inactive cushioning beads comprising at least one compressible cushioning component consisting essentially of a microcrystalline hydrocarbon wax or a natural wax, which is at least 30% by weight of the biologically inactive cushioning beads, were found useful as cushioning excipients. Such beads could minimize the damage to film-coated diltiazem pellets during compression^49^,^50^. Yuasa and co-workers studied the protective effect of 14 different excipients and were able to correlate the plastic energy percentage to the release rate *i.e*., materials that deform plastically were shown to protect the coating best^51^.

Tunon *et al*. investigated the influence of the size and the porosity of excipient microcrystalline cellulose (MCC) particles on the densification and deformation of the reservoir pellets during compaction and also investigated the impact of the same on the drug release from reservoir pellets. MCC pellets of different size (small and large) and porosity (low and high) were prepared by extrusion-spheronization or direct spheronization. Salicylic acid pellets containing MCC were prepared by extrusion-spheronization and spray coated with ethyl cellulose. Binary mixtures of drug pellets and excipient pellets were prepared in 1:7 ratio, lubricated and then compacted. Reservoir pellets were retrieved by deaggregation of the tablets. Scanning electron images revealed that the pellets were deformed and were not defragmented. Intragranular porosity of the retrieved pellets was found to be lower than the original porosity.

The deformation of the reservoir pellets was found to be strongly dependent on the properties of the excipients. The deformation of the reservoir pellets was more when compacted with small and large excipient pellets. The same was found to be less when compacted with MCC powder of small particles. The deformation of the reservoir pellets increased with decreased excipient pellet porosity or increased compaction pressure. With respect to the drug release from the reservoir pellets, the reduced size and the increased porosity of the excipient pellets had very limited effect compared to the drug release rate or profile of the uncompacted reservoir pellets. Shortest drug release time and the highest drug release from the reservoir pellets were observed on compaction with large low porosity pellets. It can be concluded from these findings that the physical characteristics of the pellet core and the protective particles represent vital formulation factors for the compaction behaviour of reservoir pellets and for maintaining the polymer coat integrity during compaction and thereby maintaining the functional property of the polymer coat^52^.

### Amount and size of the excipient particles:

The amount as well as the size of the excipient particles are also crucial for the protective effect. Lehmann and co-workers concluded that inclusion of approximately 30% of excipients in the tablet formulation filled the void space between the coated pellets, and thus separated the coatings, so that the tablets disintegrated rapidly with insignificant damage to the coatings and no notable change in drug release^3^. Palmieri and co-workers showed that the tablets consisting of maximum 40% coated granules had acceptable release profiles, using microcrystalline cellulose as the filler excipient^53^.

With respect to tablet hardness, the maximum level of drug pellets that gives tablets, which are strong enough, was found to be 40%^54^. With respect to the excipient particle size, El-Mahdi and Deasy found that excipient powder particles protected coated pellets better than excipient pellets, in which case significant damage occurred to the reservoir pellets^55^. In a study by Yao and co-workers, excipient particles smaller than 20 μm were found to protect the coating of theophylline particles irrespective of the excipient material used, while larger excipient particles increased the dissolution rate on compaction^56^. Yuasa and co-workers compacted microcapsules with mixtures of excipients including MCC of various sizes. Small MCC particles increased the dissolution rate less *i.e.,* protected the microcapsules better, than larger ones^57^.

Differences between the size as well as the density of the pellets and that of the excipient particles lead to segregation of the pellets from the powder blend^58^. The segregation also occurs frequently on the turret of a rotary compression machine due to vibration and centrifugal force. If not addressed properly, the segregation results in weight variation and content uniformity problems^59^. Ando *et al*. developed a novel one step dry-coated tablet technology (OSDRC® technology) for pellets which mainly addresses the segregation problem^60^. The technique involves three compression stages. In the first stage, a bottom outer layer is formed. In the second stage, first outer layer/core layer complex is formed and in the third stage, whole tablet containing upper outer and side outer layer is formed. The first and last layers essentially contain diluents with good formability characteristics while the core layer contains pellets. As this process involves compression of diluents and pellets in separate stages, segregation problem does not arise and therefore weight variation and content uniformity problems that occur due to segregation can be addressed by this novel technology. The technology enables the compression of pellets with very poor formability (tensile strength of ≤ 2 KPa). It also enables to prepare capsule shaped tablets containing pellets of 50% w/w or more.

### Newer approaches:

A different approach for protection that has been investigated is the layering of cushioning agents as extra coating layers to the reservoir pellets. In an investigation by Altaf *et al*. polyethylene oxide (PEO) was evaluated for its cushioning effect. The PEO was spray coated between the ethylcellulose and microcrystalline cellulose coats. The compacted PEO layered beads, on dissolution, disintegrated into individual beads with sustained release up to 8 h. It was postulated that the PEO was hydrated and formed a gel that acts as a sealant for the cracks formed in the ruptured polymer coating^61^.

The over coating of HPMC was advised to protect film coating during compression because it produces a soft and pliable film, which could behave as a cushioning excipient^62^. Chambin *et al*. investigated the effectiveness of hydroxypropyl methylcellulose (HPMC) as a cushioning agent. A thin layer of HPMC 5cp as Opadry® OY-7240 was coated over ethylcellulose coated theophylline pellets. This overcoating of HPMC was found to minimize the coating film damage during compression^63^.

In a recent study by Abbaspour *et al*., ibuprofen pellets were prepared by extrusion and spheronization with 60% ibuprofen, 27% Eudragit RS/RL (1:1), 10% Avicel and 3% PVP K30 as the basic core formula. These pellets were coated with a mixture of Eudragit RS30D and Eudragit RL30D (4:1) with 20% w/w triethyl citrate (TEC) as plasticizer at 5%, 10% and 15% w/w weight gains and subjected to dissolution study. Results indicated that sustained release of ibuprofen over a period of 24 h could be achieved with 5% coating level. Higher coating levels resulted in a very slow drug release. The ibuprofen pellets coated at 5% coating level were tableted using different filler blends consisting of Avicel, PEG and PVP XL. The effect of compression force and percent of pellets on tablet properties were also evaluated. None of these parameters were found to affect the ibuprofen release rate from their disintegrating compacts^64^. This indicates that the Eudragit coating (RS30D/RL30D (4:1); 20% w/w TEC) at 5% provided cushioning effect to the pellets and helped in maintaining the polymer coat integrity even after compression process.

## CONCLUSIONS

Compression of coated pellets is a challenging task, which needs optimization of several key variables. Out of various formulation parameters, the pellet core, polymer coating and tabletting excipients are the key variables, which need to be given more focus. The pellet core must be hard enough to withstand the compression force, yet soft enough to get deformed without brittle fracture on compression. Until recently, MCC has been known as the best core material. However, in a recent study cured Eudragit RS PO and RL PO based pellets have been found to be beneficial as core materials, particularly for the production of plastic pellets that are to be coated and compressed into tablets. The polymer coat must be flexible enough to not rupture during compression and to retain its drug release controlling nature even after compaction. Films based on acrylic polymers such as Eudragit® NE30D, Eudragit® RS and RL30D (plasticized) are very flexible and more suitable for the coating of pellets intended to be compressed into tablets. The excipient particles must preferably be similar in size and density as that of the active pellets to avoid segregation problem during compaction. Recently, OSDRC® technology has been developed which involves compression of diluents and pellets in separate stages which addresses segregation and associated problems. The polymer coat should be cushioned either by means of incorporating an excipient having cushioning properties as filler material or coating such a material over the active pellets. MCC is a well studied cushioning excipient. Beads based on waxy materials such as microcrystalline hydrocarbon wax or natural wax have been reported to have good cushioning properties. Approaches based on coating of cushioning materials such as poly ethylene oxide (PEO) and hydroxypropyl methylcellulose (HPMC) over core pellets have been investigated and were found to be fruitful in maintaining the functional coating integrity up on compaction. The pellet: excipient ratio must also be optimized to prevent polymer coat rupture, weight variation and to have tablet of sufficient mechanical properties.
